# Humidity

**Published:** 1912-11

**Authors:** 


// BUFFALO MEDICAL JOURNAL

**A Monthly Review of Medicine and Surgery**

*EDITOR*

A. L. BENEDICT.

All communications, whether of a literary or business nature, books for review and exchanges, should be addressed to the editor, **228 Summer Street, Cor. Elmwood Avenue, Buffalo, N. Y. Subscribers, advertisers and exchanges please revise address.** Please make personal or telephonic calls before 1 p. m.

THE BUFFALO MEDICAL JOURNAL will publish regularly, full transactions of any professional organization in western New York at cost. Personal notices, brief reports of society proceedings, and the like, are always welcome and will be published without charge.

Subscriptions may commence at any time. $2.00 per year in advance.

VOL. LXVIII. NOVEMBER, 1912 No. 4

*A man's greatness is shown by his treatment of the little man.*

### Humidity

For the next six months, the majority of our population will spend a large share of their time indoors, with a temperature of about the same as during the summer, while wearing about twice as much clothing. And, for the most part, they will exercise almost as much and will eat more heat-producing food. While theoretically, houses, schools and work rooms should be kept at a temperature correspondingly less than in summer, or else underwear should be changed on entering and leaving the indoor environment, most persons feel more comfortable if the summer temperature is maintained artificially and the latter device is impracticable and, by experience, unnecessary.

At the same time, commercial and entirely disinterested literature regarding the baneful effects of dryness are being scattered and careful householders are, more or less systematically, placing dishes of water to secure a proper humidity by evaporation. Ordinarily, in this climate, the out-door humidity is about 30 or 40 for a dry day in winter, 50 or 60 in summer and according to circumstances, the out-door humidity runs up to 80, rarely higher. Out-door air merely admitted to a room and warmed, gains in its capacity for holding water without condensation; in other words, loses in (relative) humidity. Thus, any heating system which does not communicate water of oxidation to the air, causes a marked drop in humidity. In spite of the admission of fresh air and the addition of water of respiration, the out-going air, though relatively dry, tends to carry off more water than enters so that the humidity gradually falls, the warm furnishings of an ordinary room being able to take up any sudden moderate excess. Thus, the indoor air will usually have a humidity of 20-40 and, with the torsion hygrometer which is only approximately accurate may even register zero.

Superficially considered, an environment in which anything stands at a tenth to a half of the natural standard, impresses one as dangerous, especially when the change is sudden and repeated. But it must be remembered that percentages in (relative) humidity greatly exaggerate the actual quantity of water present in the air since they depend largely on temperature and are based merely on the potential absorptive capacity of the air without precipitation. To speak of passing from a humidity of, say, 60 to 10, suggests something as serious as Caisson disease but it is infinitessimal as compared with stepping from air into a tub of water and out again, yet the latter change, equivalent to a leap from say 20 to hundreds of a per cent. and vice versa, is practiced with inpunity by most of us at frequent intervals.

It should, of course, be understood that in discussing in-door humidity, we assume sufficient ventilation, proper temperature, and adequate out-door exercise. The defense of dry indoor air must also fall to the ground if it can be shown that relative lack of humidity entails any alteration in gaseous content, radiant properties or anything else hygienically necessary. Unduly moist indoor air, with condensation of water upon windows usually indicates an excess of carbon dioxid while the latter, though probably not in itself harmful till such proportions are reached as interfere with respiratory osmosis, is usually attended by an excess of unmeasureable impurities of other nature. Dry indoor air may obviously be impure without showing this convenient index of contamination. But, generally speaking, radiation systems of heating allow more uniform distribution of heat, better ventilation and greater freedom both from drafts and from atmospheric impurities due to the source of heat than any other method. The ultimate issue, therefore, is whether the dryness of the air, in and of itself, is harmful.

It goes without saying that low atmospheric humidity, natural or artificial, tends to increase the loss of water from the body by insensible perspiration and respiration and hence tends to reduce elimination by the kidneys and bowel. Obviously, therefore, more water must be taken and, within reasonable limits, the more water is run through the body, the better. The person who does not compensate for the additional evaporation which takes place in dry air, will suffer in various ways but, as a matter of fact, such a person will usually follow a regimen insufficient for the physiologic processes requiring water as a vehicle, under any circumstances. Under no circumstances of humidity nor even of immersion, can the human body absorb sufficient water without drinking.

The question therefore resolves itself into this: Is the relative excess of evaporation in dry air harmful locally? For the skin, generally, it is universally conceded that perspiratory activity is beneficial up to the point at which it indicates excessive physical strain, excessive temperature, maceration, or is symptomatic of some pathologic condition. Under ordinary circumstances, urine, insensible perspiration and respiratory evaporation are approximately equal and amount to about a liter a day each—the last somewhat less as a rule. There is an impression that respiratory vapor is pulmonary and is added to the air before it is expired. As a matter of fact, it is largely inspiratory, added to the air as it passes over the nasal turbinates, nasal breathing being required partly to filter out by the nasal hairs and to catch upon mucous surfaces gross dirt; partly to warm, and partly to moisten the air before it reaches the lungs. It is conceivable therefore that the extra demand made upon the turbinate tissues may result in local lesions or that, if they fail to supply the necessary amount of moisture, the lungs may suffer. Indeed, it is not merely conceivable but a matter of actual knowledge that certain respiratory lesions require moist air as part of the therapeutics of the case. For the person of average health so far as the respiratory passages are concerned empiricism is better than theory. The writer's experience is that catarrhs of the upper and lower respiratory tract are less frequent with dry than with moist indoor air, though this may be partly due to the better control, more even distribution of heat, and possibility of more efficient ventilation without drafts by radiation methods.

The only other local ill effect which can be anticipated from dry indoor air, is evaporation from the conjunctiva. With an abundant ingestion of water and a normal lachrymal apparatus, this seems rather a far-fetched hypothesis.

Subject to revision, therefore, we advocate radiation systems of heating, perferably by electricity when the public service commission has done away with extortionate profits or wasteful methods of production. And we question whether the troublesome, usually filthy and intermittently supplied pan of water had not better be cleaned and relegated to the attic—if the steam-heated dwelling has such a place.

